# *Xenorhabdus* and *Photorhabdus* Bacteria as Potential Candidates for the Control of *Culex pipiens* L. (Diptera: Culicidae), the Principal Vector of West Nile Virus and Lymphatic Filariasis

**DOI:** 10.3390/pathogens12091095

**Published:** 2023-08-28

**Authors:** Ebubekir Yüksel, Alparslan Yıldırım, Mustafa İmren, Ramazan Canhilal, Abdelfattah A. Dababat

**Affiliations:** 1 Department of Plant Protection, Faculty of Agriculture, Kayseri Erciyes University, Kayseri 38030, Türkiye; r_canhilal@hotmail.com; 2 Department of Parasitology, Faculty of Veterinary Medicine, Erciyes University, Kayseri 38280, Türkiye; yildirima@erciyes.edu.tr; 3 Department of Plant Protection, Faculty of Agriculture, Abant Izzet Baysal University, Bolu 14030, Türkiye; m.imren37@gmail.com; 4 International Maize and Wheat Improvement Centre (CIMMYT), Ankara 06000, Türkiye; a.dababat@cgiar.org

**Keywords:** vector, vector-borne disease, biocontrol, endosymbionts, sustainability

## Abstract

Vector-borne diseases pose a severe threat to human and animal health. *Culex pipiens* L. (Diptera: Culicidae) is a widespread mosquito species and serves as a vector for the transmission of infectious diseases such as West Nile disease and Lymphatic Filariasis. Synthetic insecticides have been the prime control method for many years to suppress *Cx. pipiens* populations. However, recently, the use of insecticides has begun to be questioned due to the detrimental impact on human health and the natural environment. Therefore, many authorities urge the development of eco-friendly control methods that are nontoxic to humans. The bacterial associates [*Xenorhabdus* and *Photorhabdus* spp. (Enterobacterales: Morganellaceae)] of entomopathogenic nematodes (EPNs) (*Sterinernema* spp. and *Heterorhabditis* spp.) (Rhabditida: Heterorhabditidae and Steinernematidae) are one of the green approaches to combat a variety of insect pests. In the present study, the mosquitocidal activity of the cell-free supernatants and cell suspension (4 × 10^7^ cells mL^−1^) of four different symbiotic bacteria (*Xenorhabdus nematophila*, *X. bovienii*, *X. budapestensis*, and *P. luminescens* subsp. *kayaii*) was assessed against different development stages of *Cx. pipiens* (The 1st/2nd and 3rd/4th instar larvae and pupa) under laboratory conditions. The bacterial symbionts were able to kill all the development stages with varying levels of mortality. The 1st/2nd instar larvae exhibited the highest susceptibility to the cell-free supernatants and cell suspensions of symbiotic bacteria and the efficacy of the cell-free supernatants and cell suspensions gradually declined with increasing phases of growth. The highest effectiveness was achieved by the *X. bovienii* KCS-4S strain inducing 95% mortality to the 1st/2nd instar larvae. The results indicate that tested bacterial symbionts have great potential as an eco-friendly alternative to insecticides.

## 1. Introduction

Among blood-sucking insects, mosquitoes (Diptera: Culicidae) are the most well-known class of vectors of pathogens causing serious diseases in humans and animals [1,2]. To date, 26 mosquito species have been identified as disease vectors, 8 of which belong to the *Culex* genus [2,3,4]. *Culex pipiens* L. (Diptera: Culicidae) is one of the most abundant *Culex* spp. in Europe and a major vector of several diseases with medical and veterinary importance [3,4,5,6]. *Culex pipiens* has been linked to the transmission of the major cause of lymphatic filariasis, *Wuchereria bancrofti*, and viral diseases including Rift Valley fever, St. Louis encephalitis, and the Sindbis and West Nile viruses [2,5,6,7,8]. Additionally, as a possible vector, *Cx. pipiens* has also been associated with the Hepatitis C virus (HCV) [9,10,11]. Controlling vector mosquitoes plays a vital role in preventing vector-borne diseases and ensuring public health locally or nationwide as well as reducing nuisance mosquito populations. Of various mosquito control methods, synthetic insecticides such as organochlorine and organophosphate compounds are the most preferred control strategy [12]. However, over the last decade, the use of synthetic insecticides has begun to be questioned by many authorities due to continuous use or inappropriate application that exerts strong insecticide resistance selection pressure on mosquito populations and causes harmful effects on human health and the environment [13,14,15,16,17]. As a result, in recent years, developing environmentally safe and effective mosquito control strategies has gained importance and this has prompted many researchers to investigate various alternative methods in the control of vector mosquitoes [18,19,20]. 

Entomopathogenic nematodes (EPNs) (*Steinernema* spp. and *Heterorhabditis* spp.) and their bacterial symbionts [*Xenorhabdus* and *Photorhabdus* spp. (Enterobacterales: Morganellaceae)] are lethal parasites of insect pests and an emerging approach to controlling many economically impactful pests [21,22,23,24]. The bacterial associates of EPNs, after being vectored into a host body by infective juveniles (IJs) of EPNs, multiply using the host hemolymph as a source of nutrition and release a wide range of metabolites including toxin complexes (Tc) and immunosuppressants into the host hemolymph that is lethal to host insects [25,26,27]. Recent studies have shown that bacterial symbionts alone are capable of inducing varying levels of mortality against target insects without their nematode partners [28,29,30,31]. However, only a few studies have tested the pathogenic activity of bacterial symbionts of EPNs against *Cx. pipiens* [29]. In addition, the differences in the mortality of test insects have been generally attributed to the variation in the frequency and chemical compositions of secondary metabolites with insecticidal and immunosuppressant activities produced by different symbiotic bacteria species/strains [32,33,34,35,36]. Therefore, the screening of pathogenicity of various *Xenorhabdus* and *Photorhabdus* species/strains on the target host are of crucial importance for ensuring effective biocontrol. In this study, the mosquitocidal efficacy of cell-free supernatants and cell suspensions of different symbiotic bacteria (*X. nematophilai*, *X. bovienii*, *X. budapestensis*, and *P. luminescens* subsp. *kayaii*) was evaluated against the different development stages of *Cx. pipiens*.

## 2. Materials and Methods

### 2.1. Source of Mosquito

Different larval instars and pupae of *Cx. pipiens* were obtained from the stock culture of the Parasitology Department of the Faculty of Veterinary Medicine, Erciyes University, Türkiye [6]. The larvae and pupae were observed for one day after being transported to the Laboratory of Entomology (Erciyes University) and kept in an incubator at 25 °C, 60% RH, and a photoperiod of 14:10 h (Light:Darkness).

### 2.2. Bacterial Strains

Four bacterial strains of EPNs [*Xenorhabdus bovienii* (MZ688381), *Xenorhabdus budapestensis* (MW403817), *Xenorhabdus nematophila* (MZ688376), and *Photorhabdus luminescens* subsp. *kayaii* (MW403818)] isolated and identified in earlier studies were tested for their mosquitocidal activity in the bioassays [35]. 

### 2.3. Isolation of Symbiotic Bacteria

In order to extract the bacterial symbionts of EPNs, ten *Galleria mellonella* (Linnaeus) (Lepidoptera: Pyralidae) larvae were exposed to IJs of nematode partners of each symbiotic bacterium at 200 IJs/mL distilled water. Dead larvae were transferred to sterile Petri dishes and placed on modified White’s traps for the emergence of IJs. Approximately 500 newly harvested IJs kept in sodium hypochlorite (10% w/vol) (NaOCl) solution for disinfection were crushed manually using a sterile tissue grinder in a 1.5 mL microcentrifuge containing 1 mL of sterile PBS buffer. Subsequently, 20 μL of the suspension was inoculated on nutrient bromothymol blue triphenyl tetrazolium chloride agar (NBTA medium) [25,26,28] and maintained at 28 °C (20% RH, in darkness) for 48 h. Pure bacterial colonies were obtained by re-streaking the single colonies of phase I variants onto the NBTA plates [37,38]. The bacterial cells of purified colonies were inoculated into the larvae of *G. mellonella* using a sterile needle for each injection to confirm the pathogenicity of the symbiotic bacteria. Then, bacterial symbionts were re-isolated from the hemolymph of dead larvae and grown on the NBTA medium at 28 °C for 48 h [36,37,38].

### 2.4. Cell-free Supernatants and Cell Suspensions

A single bacterial colony of each pure culture was transferred into 250 mL Erlenmeyer flasks containing 100 mL Luria-Bertani broth (LB) (Sigma-Aldrich, USA) with a sterile loop and incubated for 6 days at 28 ± 1 °C in a shaking incubator at 150 rpm [36,37,38]. Then, sub-cultured bacteria suspensions were transferred into 50 mL sterile Falcon tubes. The supernatant and pellets were carefully separated from the suspension by centrifugation twice at 20,000 rpm for 15 min at 4 °C. The final supernatant fractions were clarified by passing through a 0.22 μm millipore filter [39,40]. The presence of bacterial cells in the filtrated suspensions was checked by streaking a drop of each suspension on the NBTA medium. The remaining bacterial cell pellets were purified by washing with sterile distilled water twice. The resulting bacterial pellets were re-suspended in 5 mL of sterile distilled water. The total number of cells was calculated by using a spectrophotometer (OD600, 600 nm) and adjusted to a final concentration of 4 × 10^7^ cells/mL by diluting with sterile distilled water [37]. The cell-free supernatants and cell suspensions were stored for one week at 14 °C prior to their use in the experiment.

### 2.5. Bioassays

Different developmental stages of mosquitoes generally occur in mixed groups in outdoor circumstances. Therefore, the susceptibility of mixed instars (1st/2nd and 3rd/4th) and pupae was tested against the cell-free supernatants and cell suspensions of four different bacterial symbionts of EPNs under controlled conditions. The experiments were performed in 24-well plates containing 1.5 mL of distilled water. One single larva of either the 1st/2nd or 3rd/4th instars was simultaneously put into each well using a plastic Pasteur pipette to avoid cannibalism [27]. As much finely ground commercial fish food as would fit on the tip of a toothpick was added to each well plate as a source of nutrition. Then, 0.5 mL of cell-free supernatant or cell suspension was pipetted into each well. Ten larvae were used for each treatment and each treatment consisted of four replicates. In the control treatments, the well plates were treated with the Luria-Bertani broth only and the same experimental procedures were followed. The well plates were maintained at 25 °C, 60% RH, and a photoperiod of 14:10 h (Light:Darkness) and checked for larval and pupal mortality daily for three days by gently poking larvae with a sterile pipette. The larvae and pupae were considered dead when no mobility was observed after several poking. To confirm bacterial infection, the hemolymph of the dead larvae and pupae was inoculated onto NBTA plates and incubated at 28 °C for 48 h. The experiment was carried out twice on different dates and mortality data from the two experiments were pooled for statistical analysis.

### 2.6. Statistical Analysis 

The arcsine-transformed data were subjected to factorial repeated measures ANOVA (RM-ANOVA) using IBM SPSS statistics (Version 29) (SPSS Inc., Chicago, IL, USA). Tukey’s multiple range tests (*p* ≤ 0.05) were performed to make multiple comparisons. Lethal times (The exposure time of different development stages of *Cx. pipiens* to cell-free supernatants and cell suspensions at which the mortality reached 50% of each tested population) (LT_50_) were calculated with probit analysis with 95% confidence for each treatment.

## 3. Results

### 3.1. Susceptibility of Cx. pipiens to Cell-Free Supernatants of Bacteria

The results showed that the mortality of *Cx. pipiens* was significantly affected by all main factors (Table S1). The 1st/2nd instar larvae were the most susceptible developmental stage to the tested cell-free supernatants, followed by the 3rd/4th instar larvae and pupae. The highest efficacies were achieved as the exposure time to the supernatants increased. Among the tested cell-free supernatants of symbiotic bacteria, the highest effectiveness was obtained by *X. bovienii* KCS-4S inducing 65, 80, and 95% mortality on the 1st/2nd instar larvae 24, 48, and 72 h after treatment, respectively. The sensitivity with the 3rd/4th instar larvae ranged between 22.5% and 62.5%. *Xenorhabdus bovienii* KCS-4S was the only strain that exhibited mortality greater than 50% on pupae (Table 1).

### 3.2. Susceptibility of Cx. pipiens to Cell Suspensions of Bacteria

The cell suspensions of the tested symbiotic bacteria were lethal to the different development stages of *Cx. pipiens* with varying levels of virulence and all variables [Symbiotic bacteria (S), Development Stage (D), and exposure time] including the interaction between S×D and S×t influenced the mortality rates of *Cx. pipiens* (Table S2).

However, although the mortality rates showed an increasing trend with rising exposure times, the cell suspension treatments had relatively low mortalities in the different development stages of *Cx. pipiens* compared to cell-free supernatants. *Xenorhabdus nematophila* E-76 was the most efficient strain causing 57.5% mortality in the 1st/2nd instar larvae, followed by *X. bovienii* KCS-4S (50.0%). However, the cell suspensions demonstrated a gradual decline in toxicity in the 3rd/4th instar larvae and pupae (Table 2).

In general, the lowest LT_50_ values were obtained in the 1st/2nd instar larvae and the LT_50_ values increased with the 3rd/4th instar larvae and pupae. The LT_50_ values ranged between 21.2 and 77.1 in the cell-free supernatant treatments while higher LT_50_ values were obtained in the cell-suspension treatments, varying between 53.4 and 74.6 h. The supernatant of *X. bovienii* KCS-4S strain was the most toxic among the supernatants of the tested bacteria and provided the lowest LT_50_ values in the different development stages of *Cx. pipiens*. However, in the cell suspension treatments, *X. nematophila* E-76 yielded the lowest LT_50_ values (Table 3).

## 4. Discussion

The bacterial symbionts of EPNs have attracted great attention in the last decade due to their biocontrol potential against a wide range of agricultural pests including insect vectors [27,41,42,43,44,45]. However, although efficient, most of these studies targeted a single development stage of the target pests [27,28,29,30,32,43]. In the present study, the toxicity of different bacterial symbionts of EPNs was evaluated against different development stages of *Cx. pipiens*. The results revealed that 1st/2nd instar larvae were highly susceptible to the cell-free supernatants of tested bacterial symbionts while 3rd/4th instar larvae and pupae showed a moderate sensitivity. These results are in line with Da Silva et al. [27], who reported 52% and 73% mortality in the 3rd/4th instar larvae of *Aedes aegypti* (Linnaeus) (Diptera: Culicidae) after treatment with *X. nematophila* and *P. luminescens*, respectively. In contrast, 98% larval mortality was reported in another study against the 3rd instar of *A. aegypti* larvae when treated with a 2 mL bacterial suspension of *Xenorhabdus ehlersii* (bMH9.2_TH) containing 10^8^ CFU/mL [28]. The discrepancy in mortality rates may be due to the differences in the amount and composition of secondary metabolites (toxins, enzymes, and proteins) produced by different species/strains of bacterial associates of EPNs, metabolites that exhibit varying levels of insecticidal and immunosuppressant activities [25,46,47,48,49]. For instance, Hasan et al. [33] reported a great variation in the secondary metabolite production of different strains of *X. nematophila* and a positive correlation between secondary metabolite production and mortality in *Spodoptera exigua* (Hübner) (Noctuidae: Lepidoptera) larvae. In another study, Fukuruksa et al. [28] tested the insecticidal activities of different *Xenorhabdus stockiae* strains on *A. aegypti* larvae, and the mortality rates ranged between 2% and 64%. Sheetal et al. [41] reported that purified lecithinase enzyme produced by *Xenorhabdus* sp. was found to be highly toxic to the 3rd instar larvae of *Culex quinquefasciatus* (Say) (Diptera: Culicidae). Similarly, Vani and Lalithambika [47] isolated an insecticidal protein that caused 93% mortality on the 3rd instar larvae of *Anopheles gambiae* Giles (Diptera: Culicidae). Although no toxic compounds were characterized in this study, the results indicate that the cell-free supernatants of tested bacteria contain highly toxic substances that can be used against *Cx. pipiens*.

This study also revealed that early instars of *Cx. pipiens* larvae exhibited a higher susceptibility to both cell-free supernatants and cell suspension solutions, which is consistent with the findings of Ünal et al. [48] and Fathy et al. [49] who reported a higher efficacy of *Xenorhabdus* and *Photorhabdus* bacteria on the early instars of *Agrotis ipsilon* (Hufnagel) (Lepidoptera: Noctuidae) and *Schistocerca gregaria* (Forsskål) (Orthoptera: Acrididae) larvae/nymph, respectively. One possible reason for this may be the differences in the immune responses of insects in different life stages. The insects counteract entomopathogens with different cellular and humoral responses. However, a number of studies demonstrated that insect immunological reactions can vary depending on the development stage of the insects [50,51,52,53]. For instance, Abdolmaleki et al. [54] indicated that late instars of *Pieris brassicae* (Linnaeus) (Lepidoptera, Pieridae) larvae, when challenged with the culture broth of *Photorhabdus temperate* subsp. *temperata* exhibited much higher phenoloxidase activity compared to early instars. This may also have played a role in the poor efficacy of cell suspension treatments against the larvae due to the activation of the innate immune system upon the detection of bacterial cells.

## 5. Conclusions

In conclusion, cell-free supernatant treatments provided higher mortality against different development stages of *Cx. pipiens* than cell suspension treatments. The highest efficacy among the tested development stages (the 1st/2nd instar and 3rd/4th instar larvae and pupae) was obtained by *X. bovienii* KCS-4S in cell-free supernatant treatments against the 1st/2nd instar larvae. Although the pupa of *Cx. pipiens* was the least susceptible stage to the cell-free supernatants, 55% mortality was achieved by *X. bovienii* KCS-4S. The results indicate that the use of the tested *Xenorhabdus* and *Photrhabdus* bacteria could be a novel approach in the biocontrol of *Cx. pipiens*. However, further studies are needed to identify, isolate, and assess the biocontrol potential of bioactive compounds that display potent insecticidal activities. 

## Figures and Tables

**Table 1:** The mosquitocidal activity of cell-free supernatants of different symbiotic bacteria on the mortality (%) of different development stages of *Culex pipiens* L. (Diptera: Culicidae) 24, 48, and 72 h post-treatment.

Stage	TPT (h)		Cell-free Supernatant * (Means ± SE)			
		Control	AVB-15	KCS-4S	MGZ-4S	E-76
L1/L2	24	0.0 ± 0.0 ^Aa^	42.5 ± 5.0 ^Ab^	65.0 ± 5.7 ^Ac^	57.5 ± 5.0 ^Abc^	45.0 ± 5.7 ^Ab^
	48	2.5 ± 5.0 ^Aa^	52.5 ± 5.0 ^Ab^	80.0 ± 8.1 ^ABc^	65.0 ± 5.7 ^Ab^	60.0 ± 8.1 ^Bb^
	72	2.5 ± 5.0 ^Aa^	72.5 ± 12.5 ^Bb^	95.0 ± 5.7 ^Bc^	85.0 ± 5.7 ^Bbc^	70.0 ± 8.1 ^Bb^
L3/L4	24	0.0 ± 0.0 ^Aa^	22.5 ± 5.0 ^Ab^	42.5 ± 5.0 ^Ac^	32.5 ± 5.0 ^Abc^	27.5 ± 5.0 ^Ab^
	48	2.5 ± 5.0 ^Aa^	37.5 ± 5.0 ^Ab^	50.0 ± 0.0 ^Ac^	55.0 ± 5.7 ^Bc^	47.5 ± 5.0 ^Bbc^
	72	5.0 ± 2.5 ^Aa^	55.0 ± 5.7 ^Bb^	62.5 ± 5.0 ^Bb^	62.5 ± 5.0 ^Bb^	62.5 ± 5.0 ^Cb^
Pupa	24	2.5 ± 5.0 ^Aa^	12.5 ± 5.0 ^Aa^	25.0 ± 5.7 ^Ab^	12.5 ± 5.0 ^Aa^	5.0 ± 5.7 ^Aa^
	48	7.5 ± 5.0 ^Aa^	25.0 ± 5.7 ^Bb^	42.5 ± 5.0 ^Bc^	25.0 ± 5.7 ^Bb^	27.5 ± 5.0 ^Bb^
	72	7.5 ± 5.0 ^Aa^	37.5 ± 5.0 ^Bb^	55.0 ± 5.7 ^Bc^	37.5 ± 5.0 ^Bb^	42.5 ± 5.0 ^Cb^

* AVB-15: *Photorhabdus luminescens* subsp. *kayaii*, KCS-4S: *Xenorhabdus bovienii*, E-76: *Xenorhabdus nematophila*, MGZ-4S: *Xenorhabdus budapestensis*, L1/L2: The first and second larval instars of *Culex pipiens*; L3/L4: The third and fourth larval instars of *Culex pipiens.* TPT: Time post-treatment. Different capital letters show statistically significant differences among exposure times for each cell-free supernatant of symbiotic bacteria (Tukey, *p* ≤ 0.05). Different lowercase letters show statistically significant differences among the cell-free supernatants of symbiotic bacteria (Tukey, *p* ≤ 0.05).

**Table 2:** The mosquitocidal activity of cell suspension of different symbiotic bacteria on the mortality (%) of different development stages of *Culex pipiens* L. (Diptera: Culicidae) 24, 48, and 72 h post-treatment (HAT).

Stage	TPT (h)		Cell Suspension * (Means ± SE)			
		Control	AVB-15	KCS-4S	MGZ-4S	E-76
L1/L2	24	0.0 ± 0.0 ^Aa^	7.5 ± 5.0 ^Ab^	27.5 ± 5.0 ^Ac^	12.5 ± 5.0 ^Ab^	25.0 ± 5.7 ^Ac^
	48	2.5 ± 5.0 ^Aa^	25.0 ± 5.7 ^Bb^	37.5 ± 5.0 ^Abc^	20.0 ± 0.0 ^Ab^	45.0 ± 5.7 ^Bc^
	72	2.5 ± 5.0 ^Aa^	37.5 ± 5.0 ^Bb^	50.0 ± 0.0 ^Bc^	35.0 ± 5.7 ^Bb^	57.5 ± 5.0 ^Bc^
L3/L4	24	0.0 ± 0.0 ^Aa^	2.5 ± 5.0 ^Aa^	17.5 ± 5.0 ^Ab^	2.5 ± 5.0 ^Aa^	22.5 ± 5.0 ^Ab^
	48	2.5 ± 5.0 ^Aa^	12.5 ± 5.0 ^Bab^	22.5 ± 5.0 ^Ab^	15.0 ± 5.7 ^Bab^	30.0 ± 0.0 ^Ab^
	72	5.0 ± 2.5 ^Aa^	22.5 ± 5.0 ^B^	35.0 ± 5.7 ^B^	22.5 ± 5.0 ^B^	35.0 ± 5.7 ^A^
Pupa	24	2.5 ± 5.0 ^Aa^	2.5 ± 5.0 ^Aa^	12.5 ± 5.0 ^Aab^	5.0 ± 5.7 ^Aa^	17.5 ± 5.0 ^Ab^
	48	7.5 ± 5.0 ^Aa^	12.5 ± 9.5 ^ABa^	22.5 ± 5.0 ^Aa^	15.0 ± 5.7 ^ABa^	22.5 ± 5.0 ^Aa^
	72	7.5 ± 5.0 ^Aa^	20.0 ± 8.1 ^Aab^	35.0 ± 5.7 ^Bb^	20.0 ± 8.1 ^Bab^	32.5 ± 5.0 ^Ab^

* AVB-15: *Photorhabdus luminescens* subsp. *kayaii*, KCS-4S: *Xenorhabdus bovienii*, E-76: *Xenorhabdus nematophila*, MGZ-4S: *Xenorhabdus budapestensis*, L1/L2: The first and second larval instars of *Culex pipiens*; L3/L4: The third and fourth larval instars of *Culex pipiens.* TPT: Time post-treatment. Different capital letters show statistically significant differences among exposure times for each cell-free supernatant of symbiotic bacteria (Tukey, *p* ≤ 0.05). Different lowercase letters show statistically significant differences among the cell-free supernatants of symbiotic bacteria (Tukey, *p* ≤ 0.05).

**Table 3:** Comparison of lethal times (LT_50_) of cell-free supernatants and cell suspensions of symbiotic bacteria on different development stages of *Culex pipiens* L. (Diptera: Culicidae).

Probit Analysis *	Cell-Free Supernatants				Cell Suspension			
	AVB-15	KCS-4	MGZ-4S	E-76	AVB-15	KCS-4	MGZ-4S	E-76
L1/L2								
*n*	40	40	40	40	40	40	40	40
X^2^	28.216	5.950	18.315	25.766	36.354	44.302	48.874	32.618
df	2	2	2	2	3	3	3	3
Slope ± SE	2.6 ± 0.3	3.0 ± 0.3	2.4 ± 0.3	2.4 ± 0.3	2.8 ± 0.2	1.8 ± 0.1	2.6 ± 0.2	1.8 ± 0.1
LT_50_ (h)	34.4	21.2	24.1	31.4	66.8	56.3	67.3	53.4
L3/L4								
*n*	40	40	40	40	40	40	40	40
X^2^	41.837	37.080	31.317	31.130	50.623	57.267	48.853	62.330
df	2	2	2	2	3	3	3	3
Slope ± SE	3.3 ± 0.3	2.3 ± 0.3	2.8 ± 0.3	3.1 ± 0.3	4.1 ± 0.3	2.3 ± 0.1	3.9 ± 0.3	2.0 ± 0.1
LT_50_ (h)	49.5	35.7	39.5	43.3	74.2	65.9	73.5	63.2
Pupa								
*n*	40	40	40	40	40	40	40	40
X^2^	21.031	5.695	10.150	9.051	55.037	47.731	62.989	62.343
df	2	2	2	2	3	3	3	3
Slope ± SE	3.0 ± 0.3	2.2 ± 0.3	2.6 ± 0.3	3.9 ± 0.4	4.1 ± 0.3	2.5 ± 0.2	3.4 ± 0.2	2.3 ± 0.1
LT_50_ (h)	70.3	51.6	77.1	68.8	74.6	66.9	73.4	66.7

* AVB-15: *Photorhabdus luminescens* subsp. *kayaii*, KCS-4S: *Xenorhabdus bovienii*, E-76: *Xenorhabdus nematophila*, MGZ-4S: *Xenorhabdus budapestensis*, L1/L2: The first and second larval instars of *Culex pipiens*; L3/L4: The third and fourth larval instars of *Culex pipiens.*

## Data Availability

Data generated in this study are available upon reasonable request to the corresponding author.

## Supplementary Materials

The following supporting information can be downloaded at: https://www.mdpi.com/article/10.3390/pathogens12091095/s1, Table S1: Summary of statistical analysis of the mortality data of different development stages of *Culex pipiens* L. (Diptera: Culicidae) after treatment with cell-free supernatants of different symbiotic bacteria; Table S2: Summary of statistical analysis of the mortality data of different development stages of *Culex pipiens* after treatment with cell suspensions of different symbiotic bacteria.
