# Supplementary material for: Xenorhabdus and Photorhabdus Bacteria as Potential Candidates for the Control of Culex pipiens L. (Diptera: Culicidae), the Principal Vector of West Nile Virus and Lymphatic Filariasis
Source: Pathogens. 2023 Aug 28;12(9):1095. doi: 10.3390/pathogens12091095 (PMC10537861; doi:10.3390/pathogens12091095)
Supplement: Supplementary file 1 [file pathogens-12-01095-s001.zip › pathogens-2493764-supplementary.pdf]

Table S1. Summary of statistical analysis of the mortality data of different development stages of *Culex pipiens* L. (Diptera: Culicidae) after treatment with cell-free supernatants of different symbiotic bacteria.

Sources*	df	F-Value	P-Value
Symbiotic bacteria (S)	3	275.235	<0.001
Development Stage (D)	2	272.784	<0.001
S*D	6	24.069	<0.001
Error-1	45		
Exposure Time (t)	2	351.908	<0.001
D*t	4	0.813	0.520
S*t	6	10.764	<0.001
D*S*t	12	1.526	0.108
Error-2	90		

*Tukey, $P \leq 0.05$; df: Degree of freedom.

Table S2. Summary of statistical analysis of the mortality data of different development stages of *Culex pipiens* after treatment with cell suspensions of different symbiotic bacteria.

Sources*	df	F-Value	P-Value
Symbiotic bacteria (S)	3	105.430	<0.001
Development Stage (D)	2	56.092	<0.001
S*D	6	207.778	<0.001
Error-1	45		
Exposure Time (t)	2	186.986	<0.001
D*t	4	3.336	0.014
S*t	6	3.865	<0.001
D*S*t	12	1.277	0.229
Error-2	90		

*Tukey ($P \leq 0.05$); df: Degree of freedom.
